# The Perfidy of Stigma Experienced by the Palliative CHBC of Kanye in Botswana

**DOI:** 10.4103/0973-1075.63132

**Published:** 2010

**Authors:** Simon Kangethe

**Affiliations:** Department of Bachelor of Business Administration (BBA), Centre for Continuous Education (CCE), University of Botswana, Botswana

**Keywords:** Care giving, Community home based care (CHBC) program, Palliative caregivers, People living with HIV/AIDS (PLWHAs), Stigma and discrimination

## Abstract

**Background/Aim:**

To explore and assess the magnitude of stigma and its impact to palliative care giving.

**Objective:**

To involve the palliative caregivers in exploring the impact of stigma in their care giving.

**Materials and Methods:**

The study was exploratory in nature and used attracted qualitative design and interviewed 82 palliative caregivers in 10 focus groups using an interview guide as a data collection instrument, and five CHBC nurses on one-to-one in-depth interviews, still guided by an interview guide that differed only slightly with the one for the caregivers.

**Results:**

The study findings revealed that stigma and discrimination was immensely perfidious due to: (1) Discrimination against caregivers by the service providers, especially at the Kanye referral hospital; (2) Refusal of youth to help the elderly caregivers; (3) Shunning of government assistance packages by caregivers and their clients; (4) Caregivers secretly taking away their clients to faraway places for assistance; (5) Caregivers and their clients turning to alternative therapies from the traditional healers; (6) Caregivers and clients having inadequate assistance.

**Recommendations:**

We recommend strong anti-stigma education and campaign by the government, non-governmental organizations (NGOs) and all the civil society bodies and campaigners.

## INTRODUCTION

Palliative caregivers refer to family caregivers who stay with CHBC clients (HIV/AIDS and other chronically ill persons) to offer palliative care.

Palliative care is the care that is carried out, not to heal, but to relieve pain, distress, psychological loss, feeling of worthlessness and anxiety and which, gives hope, dignity, esteem, happiness, and inculcate the feelings that all will be well to a sick person.

Perfidy is a negative and undesirable state of affairs.

Increased HIV/AIDS statistics in Botswana and their association with the state of stigma prompted the researcher to go to the ground to assess and explore the situation dynamics with the hope of recommending robust interventions to contain the situation. The researcher, working as an HIV/AIDS advisor in the area of research for five years (2001-2005), was concerned with unofficial community complaints on the ground that CHBC clients were not adequately handled due to stigma amid other challenges. It was therefore necessary for him to institute research to authenticate these subjective claims whose empirical results could help chart the way forward to address the quagmire.

Botswana is one of the countries hardest hit by the epidemic. It ranks second in the world’s HIV/AIDS prevalence global ranking. While results from the Botswana AIDS Impact Survey II[1] indicate a national HIV prevalence of 17.1% with Chobe (29.4%), Francistown (24.6%), Selibe Phikwe (23.3%) leading other districts.[2,3] Botswana AIDS Impact Survey iii (BAIS III), conducted in 2008, shows an increase to 17.6%.[4] The presence of AIDS has had devastating national effects to the economy. It has caused Botswana to slip 51 places down the Human Development Index (HDI) rankings from an impressive 71 in 1996 to 122 in 1999/2000, putting the four decades of successful human and economic development pace in reverse gear. While the country of Botswana grapples to put interventions in place to address the effects of HIV/AIDS, stigma and discrimination have continued to pose as threat and major stumbling block.[5,6]

According to Festus Mogae, former President of the country of Botswana, stigma and discrimination surrounding the HIV/AIDS disease remains one of the greatest barriers to the implementation of various care and prevention strategies.[7] Kalanke[8] indicates that people living with HIV/AIDS (PLWHAs), because of fear associated with stigma and discrimination of HIV/AIDS, resort to using and adopting alternative coping strategies such as avoidance, denial and secrecy. This has had serious repercussions of negatively affecting the rate of consumption of services meant to help and cushion the effect of the virus to those who are affected. This has seen programs like prevention of mother to child transmission (PMTCT) suffering overtime, with eligible beneficiaries refusing to test in time and in sufficient numbers, preferring to keep their status secret for fear of discrimination, stigmatization and even physical abuse.[9]

## MATERIALS AND METHODS

### Research design

The study has used qualitative design to seek and explore the thought process, feelings and attitudes of the palliative caregivers towards their contribution to care giving generally and assessing other challenges besetting care giving such as stigma and discrimination.[7,10]

### Sample selection criteria and procedure

The study has used convenient / purposive sampling and included all the 140 registered palliative primary caregivers as well as all the four CHBC nurses and their coordinator (who manages the program) as they appeared in the CHBC register. Eighty-two (59%) registered primary caregivers turned up for 10 focus group discussions. All the nurses turned up for the interviews.

### Research instruments

The study used two separate but almost similar interview guides, one to steer 10 focus group discussions and the other to facilitate one-to-one interviews with the nurses. The first section of the instruments captured the respondents’ profile data such as age, gender and education. The instruments had, however, been tested and refined during the pilot study to improve its validity and reliability, and hence reducing data bias.

### Research domain

The study was done in December, 2005, and January, 2006, and interviewed palliative caregivers and their nurse supervisors of the Kanye CHBC program of Kanye village. The village is about a 100 kilometers from the city of Gaborone; with more than 40,0000 by the 2001 census,[11] it is well endowed with five clinics and two health posts, and a bigger Seventh Day Adventist (SDA)-run referral hospital.

### Data analysis, interpretation and bias reduction

Data analysis was done using content thematic analysis. Both sets of information from the 10 focus group discussions with primary care givers and one-to-one interviews with the nurses were taped and then transcribed. The crude data from the field was sorted and reduced to manageable categories, themes and sub themes. To effect data bias minimization and therefore increase data reliability and validity, results from the pilot study that preceded this main study formed baseline data. Double translation of the instruments was also done. However, to further strengthen data reliability and validity, the four nurses and their coordinator answered more or less the same questions with the primary caregivers. The two interview guides or the instruments only differed slightly and the collected sets of information confirmed, corroborated and cross checked each other.

### Ethical and legal requirements

To ensure that the study was grounded in legal and ethical environments, all appropriate legal and ethical issues were taken into consideration: informed consent, maintenance of confidentiality and anonymity and adequate debriefings and consultations before study kick off. All the respondents were treated with due respect to maintain their integrity and their human rights.[12] The researcher had complied with all the research permit application procedures from the Department of Research and Development Committee Board (HRDC) and therefore given a research permit. The researcher had to write a letter to the Council secretary requesting to be allowed to get into the field. Permission was out rightly given.

## RESULTS

### Profile of the volunteer caregivers

#### Age, gender and educational dimension of the caregivers

Study findings confirmed that the youngest caregiver was 18 while the oldest was 85 years old. Forty-six caregivers (56%) were older (50 years and above), with 28 (34%) being 60 years and above. The study revealed that most caregivers especially those above 60 years were poor and physically not strong enough to stand the care giving demands, with the state of stigma besetting community participation to help these caregivers. However, 88% of the caregivers confirmed they were poor and had no any income to support themselves with. Their poverty was demonstrated by some caregivers breaking into tears as they explained the economic and social environments in which they performed care.

On literacy, 74% of the caregivers had either never been to school or had only primary level education, with only five per cent with tertiary education. Illiteracy was found to contribute to low care productivity and poverty. This was psychologically disenabling as most of the lowly educated caregivers who were also elderly had problems of accessing education on care giving such as following the medical and hygiene protocol, and following the disease progression of their clients. On gender, findings indicate that the program faced serious gender skewed dimension with 80 caregivers (98%) being women and two (2%) caregivers being men. Since women have other domestic chores at their homes, care giving presented a state of burden.

#### Caregivers discriminated by healthcare providers

More than half of the study respondents expressed dissatisfaction from the Kanye referral hospital nurses who refused to give them protective clothes to wash and attend to their clients, while they themselves used them. The caregivers perceived this as discrimination. They indicated that communities stigmatized their clients and them, but they least expected the same treatment from the service providers. The following comments on stigma and discrimination were made by caregivers.

“The communities largely stigmatize our clients and us. Care giving becomes very demoralizing”

“The staff at Kanye referral hospital discriminate us by refusing to give us the protective clothing to handle our clients, while they themselves use them”

#### Caregivers fear stigma

Majority of the Kanye caregivers indicated that due to stigma and discrimination, some caregivers who had HIV/AIDS clients did not want to disclose that their clients were infected with the virus. This closed all the avenues of any possible assistance nearby, with some preferring to go for assistance far away from the client’s village. The following comments were made on the clients’ access to the help they needed:

“Some caregivers go to seek assistance far from Kanye. They do not want their clients’ status to be known by neighbors and other community members”

“Sometimes clients do not get medication or assistance in time. Money to travel to farther places of assistance may not be there. Stigma is indeed a big challenge”

#### Stigma dwindling and changing from open to latent one

About a third of the caregivers indicated that although stigma posed a huge challenge to care giving and the HIV/AIDS campaign generally, the state of stigma was dwindling and undergoing drastic changes, with people no longer stigmatizing the clients openly (open stigma), but doing it in a hidden state (latent state). This, they indicated, could be a pointer that stigma would dwindle completely with time.

“Open stigma is now gone. We now experience latent one”

#### Poor utilization of government assistance programs due to stigma

Over half of the Kanye study respondents confirmed that some community members spread rumors about their AIDS clients and themselves (caregivers). This they said increased fear prompting some to hide their clients’ statuses and disease environment from the community and therefore impede any forthcoming government assistance. Some caregivers confirmed their knowledge that some of the caregivers who were financially endowed took their clients to private practitioners away from the village to avoid being publicized and stigmatized through being seen accessing community home-based services locally.

#### Caregiver/client neglect

Study findings in Kanye confirmed that families, relatives and communities in general did not adequately assist in care giving. This resulted to some caregivers being left alone to handle care giving. They also indicated that due to impact of stigma, some clients were neglected and abandoned. An 18-year-old palliative caregiver who was a university leaver, abandoned by the brothers and sisters to take care of her ailing father, broke into tears as she tried to explain the stressful and agonizing moments she was undergoing, battling all alone with care giving that she was neither comfortable with, nor competent with.

“I cannot get time togoand look for a job as I’m left alone at home caring for my father. They all (brothers and sisters) went for good leaving me to struggle with care giving (breaks into tears)”.

#### Stigma drives caregivers to look for alternative therapies

Kanye focus group discussions revealed that some palliative caregivers never registered their clients to the CHBC program and did not want any communication with the government assistance due to stigma. Caregivers further indicated that a few caregivers and their clients who were also in the government assistance packages, upheld strong faith in the healers and were accessing their assistance, though secretly, due to fear of being persecuted by/or being denied the government assistance in the event that their secret affiliation were to be discovered by health personnel. Caregivers also indicated that inadequate medical staff and their treatment especially at the Kanye referral hospital, made some caregivers to seek the services of the traditional healers.

#### Stigma discourages youth to offer care giving assistance

Study findings in Kanye confirmed that the youth were shunning helping the caregivers in their care giving tasks due to stigma, especially to carry the clinical waste to the clinics. This is because the red plastic bags used to carry the waste were notable and stigmatized by communities. Due to inadequate transport facility to facilitate the collection of the waste from the caregivers’ homes, it was caregivers’ duty to take the clinical waste to the nearby clinics. This meant that clinical waste had to be taken on foot to the clinics by the palliative caregivers. Their attempt to have the youth assist them was usually failing. Some had the following to lament:

“Our children are refusing to help us carry the clinical waste to the clinics. This is too much for us at our age”

## DISCUSSION

Atta and Fidzani[13] indicate that over 50% of caregivers in most of the Botswana CHBC program are old and poor women who may not be able to follow the hygiene protocol in the care process. It would seem that care giving responsibilities in Botswana like many other developing countries fall in the hands of the very elderly while the young and the knowledgeable have shunned caring duties due to stigma among other factors.[14,15] The government and care managers need to increase community education and advocacy to attract the younger and the educated to share in the caring duties. Knowledgeable people are also in a position to understand the disease well and help to reduce and demystify the state of stigma.

The Kanye study indicates predominance of women over men. According to feminists such as Kelesetse, this presents societal exploitation to women and immensely contributes to feminization of poverty.[16,17] The government, civil society bodies and HIV/AIDs campaigners need to increase education and advocacy on engendering the caring roles in all segments of the society.[17] Kanye study respondents were of low educational status. A similar research undertaking by Phorano, Nthomang and Ngwenya[18] in Maun and Kweneng found similar results, with 33% of the caregivers studied having lower primary education.

The phenomenon of palliative caregivers and their HIV/AIDS clients being discriminated by the service providers is not uncommon in many care settings, Botswana not withstanding. While treatment in Kanye referral hospital sent waves of dissatisfaction among the palliative caregivers, UNAIDS[19] confirms reports revealing individuals living with HIV/AIDS being discriminated against by the health care system, with withheld treatment being one of the common aspect. UNAIDS[20] also quotes some medical doctors in Bangalore in India who openly stigmatized HIV positive persons by labeling them as individuals with “bad habits” of visiting prostitutes, of smoking and drinking and for being promiscuous. In the 2005/2006 study by this author, some caregivers felt discriminated when some caregivers were given some incontinent sheets by the clinic nurses, while others were denied.[21]

The presence of stigma and discrimination leads to denial. Denial is a psychological and a coping defense mechanism that makes the patient and the palliative caregiver fail to recognize and acknowledge the reality of the situation.[22] In a study by Khan and Stegling[23] in Kweneng, all the HIV/AIDS clients they interviewed denied being HIV positive and thus shunned the government welfare assistance packages such as food basket. This was a big drawback to care giving as most of the PLWHAs in the country are needy and necessitating help from the well-wishers and other help systems.[24] A report from Maokane village (part of the Southern District) indicated that due to stigma, clients were shunning the vegetables they were given by communities to improve their nutritional status.[25]

Kanye research respondents indicated that the state of stigma was dwindling fast, and changing from being open stigma to being latent one. In Nyorosi East ward of Kanye village (study area), for instance, stigma had taken a dramatic dimension with stigma being only subjected to those who failed to take good care of their clients.[19] BONEPWA’s study[26] on stigma in Tsabong reinforced the above observation that the state of stigma was dwindling fast, and had changed state from open to latent.

Stigma has been disastrous to the consumption of government assistance services. According to Mukamaambo and colleagues,[27] community home based care program has not well met its objectives because patients and their caregivers hate to be associated with it openly. But Kalanke[8] blames the health administration and government in general for increasing stigma by making a fertile environment for stigma by serving the PLWHAs and their caregivers separately from the other clients in the health facilities. According to Kalanke’s study participants, this was tantamount to making communities see PLWHAs as different from them, and therefore be made to delineate themselves from any person living with HIV/AIDS. This however reinforced stigma and discrimination.

This author believes that if efforts could be made by all, especially the health personnel to show everybody that HIV/AIDS is a normal disease, with no any difference from other diseases, this could be one of the turning points to kill and demystify stigma.

Stigma also reinforces neglect of both the clients and the caregivers. In Kang‘ethe’s 2004 study in Kanye, some evidence indicated that that some clients were left and abandoned the whole day hungry and despondent.[28] Other studies done in Kweneng in Botswana on care giving by Khan and Stegling,[23] found that caregivers felt unsupported by their families, relatives and community at large, while findings in Zimbabwe on care giving suggest that care programs are not supported by their communities, stigma being one of the reasons.

Stigma was also found to make caregivers and their clients look for the services of the traditional healers. This is because the services of the traditional healers have not been associated with stigma like those of modern health institutions. This made possibilities that people living with HIV/AIDS combined the two medication systems which may be harmful to the working of the ARV and therefore undermine one’s immunity system.[29] With the magnitude of HIV/AIDS in Botswana coupled with an environment of inadequate human resource to face the epidemic, the role of traditional healers need to be revisited, strengthened through training, mutual consultation and collaboration, with a possibility of integrating their services into the modern health system.[29–31]

The presence of stigma has especially affected the youth who have refused to help their grandparents and their mothers who volunteer to care. Available literature indicates that due to stigma of carrying the red plastic bags in Kanye, with the youth refusing to help their elderly grandmothers who care, it is not uncommon for some bags to be thrown into the nearby bush where animals such as dogs could open the content, the act posing pollution and environmental degradation.[32,33] This also poses challenges to the environmental campaign of a litter free generation.[32,34] According to Botswana Millennium Development Goals, Botswana will by 2016 have adequate environmental education and awareness necessary to reduce the level of environmental contamination and achieve sustainable development.[7,35] Mokgwaru,[36] in his research on health hazards associated with unskillful collection and disposal of clinical waste found that it’s a source of both environmental damage and pollution and can predispose the community to the epidemic.

## CONCLUSIONS

Stigma has been found to negatively affect the care giving quality and effectiveness. The environment, providing a fertile ground for stigma and discrimination to proliferate needs to be obliterated. Government, NGOs and all civil society organs need to work hand in hand to strengthen anti-stigma campaign across the width and breadth of the country. Involvement of persons living with the virus in the campaign frontline would be pivotal and critical, and their involvement is likely to pay great dividends in the campaign.
